# Opening Speech of Mr. James

**Published:** 1852-04-01

**Authors:** 


### OPENING SPEECH OF MR. JAMES.

Mr. James, Q.C., addressed the jury in a speech which lasted four hours in the delivery, on behalf of Mrs. Cumming, and with the view to show that that lady was in a sound state of mind, and quite capable of taking care of herself and of managing her pecuniary affairs. The learned counsel stated that the present proceedings had been instituted by the same parties—namely, the sons-in-law and the daughters of Mrs. Cumming—who issued the commission of lunacy against her in 1846, and who then withdrew that inquiry on an arrangement being made, by which they obtained a portion of the property of that unfortunate lady. The sole object of the whole proceedings was to get possession of her property, and not to give protection to her person. Her sons-in-law, Mr. Ince and Mr. Hooper, were the prime movers in the matter. They originated the inquiry of 1846. By the arrangement then made they consented to treat Mrs. Cumming as a person of sound mind. It was, however, subsequently found by the friends of Mrs. Cumming, that an arrangement had been entered into in ignorance of the real nature of the interest she possessed in the property, it being at the time supposed that she only had a life estate, whereas it was discovered that she was possessed of the fee simple. Under the advice of counsel she repudiated the agreement, and her deeds and papers having at the time of the arrangement been given up to the other parties, she brought an action to regain possession of them, in which action she succeeded. In 1848 her son-in-law, Hooper, and his wife, filed a bill to compel her to elect how she would dispose of a certain portion of her property, the result of which was, that Hooper got 50*l.* a-year settled upon him. The jury would observe that in all these transactions Mrs. Cumming was treated as a sane person; and yet in 1851 the very same persons who had entered into these arrangements with her revived the inquiry as to her state of mind, and obtained the present commission, founding it almost entirely upon the same evidence which had failed them in 1846. The condition of this lady deserved the commiseration of the jury. She was carried off to a lunatic asylum in May, 1846, and there imprisoned till the August following; during her confinement her husband died, unseen and unvisited by her, while she herself was left unvisited by either of her daughters, even to inform her of her husband's and of their father's death. Was not this a sufficient ground of dislike to her children? When the inquiry of 1846 was instituted at the Horns Tavern, Mrs. Cumming was unaided by a single friend, and had not accident brought Mr. Haynes to her assistance, she might have been in a lunatic asylum to this hour. Mr. Haynes had occasion to call on Mr. Barlow, who was the commissioner inquiring into Mrs. Cumming's case; and, seeing that Mrs. Cumming was without any legal adviser, he suggested to the commissioner that the inquiry ought to be postponed. It was adjourned accordingly; and the result was, the arrangement which had been so often referred to. Mr. James then gave a history of Mrs. Cumming's movements from that time (1846) up to the present hour. The only portion of that period to which the case of the promoters of this inquiry referred was from October, 1850, to January, 1851, during which time Mrs. Cumming was under the control of Mary Rainey and Eleanor Hickey. The prominent ground on which the other side rested their case was the absence of all natural affection on the part of Mrs. Cumming towards her daughters. He admitted that in some cases that fact would be a proof of mental disease; but if there existed a cause for such aversion, the mere *quantum* of that cause was immaterial; however apparently inadequate it might be, it was no proof of insanity. What had been the conduct of her children towards her? She had never liked Mr. Ince, though she was on terms of affection with Mrs. Ince up to the marriage of her other daughter with Mr. Hooper, which had been promoted by Mrs. Ince. That marriage gave Mrs. Cumming the greatest possible offence, and from that time she took a dislike both to Mrs. Ince and Mrs. Hooper. The learned counsel next adverted to the fact of Mrs. Cumming having been apprehended for perjury, and briefly stated the circumstances connected with that part of the case. A Mr. Ebenezer Jones, living at Newport, having seen in the newspapers an account of the proceedings against Mrs. Cumming at the Horns Tavern, wrote an anonymous letter, stating that he had known Mrs. Cumming for many years, and could prove that she was a person of acute mind and of sound intellect, and he voluntarily made an affidavit to that effect. Having on some occasion received a portion of Mrs. Cumming's rents, he assumed the character of general agent to that lady, but on Mrs. Cumming making an affidavit in Chancery in some proceedings connected with the sale of a part of her property, it was necessary she should repudiate the agency of that person, and she did so. This was made a ground of charge against her, and she was arrested, at the instance of Ebenezer Jones, for perjury. It could be shown that Jones was in communication with Mr. Ince and Mr. Hooper, and that the whole affair was a conspiracy on their part, aided by Eleanor Hickey. The charge was heard before Mr. Gilbert A'Beckett, the magistrate, who immediately dismissed it. Mr. Ince made two affidavits, but in neither of which did he deny a participation in this affair. It was true Mr. Hooper did in his affidavit deny any participation in it, but he (Mr. James) should be able to show that that denial was false. These affidavits were filed on the present commission being moved for. This system of persecution would account for Mrs. Cumming assuming the name of Cleveland, and for her so frequently changing her place of residence. There was reason to believe that the object of arresting Mrs. Cumming on a charge of perjury was to put her on her trial at Newport, in Wales, and then to get her acquitted upon the ground of insanity; the parties, not having succeeded in their former commission, thought they might probably, by such a manœuvre, get the whole of her property vested in them. The case, however, having been dismissed, what did Ebenezer Jones do? Urged on by Ince and Hooper, he preferred a bill of indictment for perjury at the Monmouth Assizes against her, but the bill was ignored. Would not these proceedings account for much of the conduct of Mrs. Cumming? This took place on the 27th of March, 1851. Having removed from Stamford-street to Worthing, she then went to Brighton, and there resumed her own name. She lived at Brighton in quietness and peace, hurting no one and interfering with no one. But she was not permitted to enjoy that state of repose long; for Mr. Turner then appeared upon the scene, and the jury had heard in what manner this poor lady was dragged from her house and taken to the Effra-hall Asylum, by the agency of that gentleman, instigated by her never-ceasing persecutors, Ince and Hooper. The learned counsel then took a review of the evidence which had been adduced in support of the commission, and, having commented upon it at considerable length, he proceeded to state the nature of the evidence which he intended to bring forward to rebut the charge of insanity against Mrs. Cumming. He proposed to show what had been the conduct of Captain Cumming towards his wife—that he was violent in temper, coarse in language, and cruel in behaviour towards her; and that if (as unfortunately he could not deny) she was occasionally betrayed into language and conduct unbecoming a lady, it was to be attributed to the contamination of his society. He squandered her fortune to pay the debts he had incurred. His moral deportment towards his female servants was of the basest character. He had more than one affiliation made upon him; it was therefore no delusion for this unhappy lady to say that her property was wasted to support his bastards. Indeed, the life which Mrs. Cumming led with him was one of unmitigated and uninterrupted misery. Having spent her fortune, and having abused her thus, were the jury to say that it was a proof of insanity that she should give way to violence of conduct and of language? Why, he would ask, was she taken to York-house Asylum? She had done no wrong to any human being; she had committed no act of violence; she had disposed of no property irrationally, or to the deterioration of her own rights; she was living harmlessly in her own dwelling, and at the very hour of her being violently taken away she was in the act of teaching a little girl her lesson. Upon the instigation of Mr. Ince, though nominally by her husband, who, before his death, regretted that he did not expire in the arms or the presence of his wife, this lady, so living and so conducting herself, was, on the certificate of a Mr. Wilmot, consigned to a lunatic asylum. The commission having been withdrawn, she went to Mrs. Hutchinson's, a most respectable person, and, as Mrs. Cumming herself stated, her only friend. She had been a musical instructress, and her husband was an engineer. Her going to the Hutchinsons had been objected to; but where was the unhappy lady to seek refuge? Her husband was dead, her home was gone, and she was persecuted by her children; where then was she to go? She was without money; for notice had been given to Sir Charles Morgan not to complete a purchase of a portion of her property in Wales, which he had contracted to do, and her tenants had been warned not to pay her any rent. From first to last her property was kept from her. After following Mrs. Cumming to Wales and to other places, where her conduct was uniformly that of a sane person, Mr. James read a letter, dated September 5, 1851, addressed to Mr. Haynes by the secretary of the lunacy commissioners, in consequence of an application by Mr. Turner that they would examine Mrs. Cumming. The letter was to the effect, that after considering the evidence of Dr. Barnes and Dr. Caldwell, and the communication made to them by the relatives of Mrs. Cumming, the commissioners had no reason to believe that Mrs. Cumming was not a free agent, and therefore they did not think it was within their province to take any further proceedings in the case. The learned counsel next called the attention of the jury to the opinion of the medical witnesses. In consequence of the proceedings adopted by Mr. Ince and Mr. Hooper, it became necessary that Mrs. Cumming should take measures in her own defence; and accordingly Dr. Forbes Winslow was directed by the Lord Chancellor to examine that lady and make his report on her case. The report was made on the 8th of December, 1851. The learned gentleman here read the report, which will be found at the end of the trial. Without saying a word of adulation to Dr. Winslow, he could not help remarking that his report was the most able and elaborate that had ever been filed in the Court of Chancery. The learned counsel then stated that he should call Dr. Caldwell, Mr. Hodding, Dr. Hale, Dr. Barnes, Dr. Conolly, and other medical gentlemen, as witnesses, who would all declare their opinion that Mrs. Cumming was perfectly of sound mind and capable of managing her own affairs. In reference to Mr. Haynes, the mention of whose name he considered to be a mere episode in the case, he should, he said, abstain from all remark. There might be a subsidiary inquiry hereafter, and no doubt that gentleman would be quite prepared to defend himself. He had, to the best of his ability, discharged his duty to Mrs. Cumming, and he knew she would have her case fairly and impartially considered. They had had the opportunity of examining that lady, and would form their own judgment upon what they had heard and observed. They would not suffer their minds to be influenced by mere eccentricity or a fondness for animals on the part of the lady in determining upon the soundness or unsoundness of her mind. It was easy to make the charge of madness against the wisest of men. "Festus said with a loud voice, 'Paul, thou art beside thyself, much learning hath made thee mad;' but he said, 'I am not mad, most noble Festus, but speak forth the words of truth and soberness.'" Socrates was charged as mad; Galileo was charged as mad, because he retired to his tower to watch the stars; and Mr. Pettigrew had told them that to believe in the ordeal of touch was madness. The charge was easily made against any person of eccentric habits; but he did implore them to look with caution at the facts that were to guide their judgment, to remember that this unfortunate lady had been literally hunted for mad, scourged for mad, whipped for mad, persecuted as mad. But he relied on them to declare by their verdict that she was not mad, though there were those who were anxious to make her so. It might be of little moment, during the few last days of her life, whether they pronounced her mad or not, for their verdict might be whispered into the ears of death, yet to the public at large the question would be of vast importance. He therefore called upon them to pause before any twelve of them pronounced this unhappy lady to be of unsound mind, as was prayed on behalf of the promoters of this commission.

